# *Staphylococcus aureus* in Some Brazilian Dairy Industries: Changes of Contamination and Diversity

**DOI:** 10.3389/fmicb.2017.02049

**Published:** 2017-10-24

**Authors:** Karen K. Dittmann, Luíza T. Chaul, Sarah H. I. Lee, Carlos H. Corassin, Carlos A. Fernandes de Oliveira, Elaine C. Pereira De Martinis, Virgínia F. Alves, Lone Gram, Virginie Oxaran

**Affiliations:** ^1^Department of Biotechnology and Biomedicine, Technical University of Denmark, Kongens Lyngby, Denmark; ^2^Faculty of Pharmacy, Federal University of Goiás, Goiânia, Brazil; ^3^Faculty of Animal Science and Food Engineering, University of São Paulo, Ribeirão Preto, Brazil; ^4^Faculty of Pharmaceutical Sciences of Ribeirão Preto, University of São Paulo, Ribeirão Preto, Brazil

**Keywords:** *Staphylococcus aureus*, dairy industry, food safety, MLST, antibiotic resistance, staphylococcal enterotoxin, Brazil

## Abstract

*Staphylococcus aureus*, a major food-poisoning pathogen, is a common contaminant in dairy industries worldwide, including in Brazil. We determined the occurrence of *S. aureus* in five dairies in Brazil over 8 months. Of 421 samples, 31 (7.4%) were positive for *S. aureus* and prevalence varied from 0 to 63.3% between dairies. Sixty-six isolates from the 31 samples were typed by Multi-Locus Sequence Typing to determine if these isolates were persistent or continuously reintroduced. Seven known sequence types (STs), ST1, ST5, ST30, ST97, ST126, ST188 and ST398, and four new ST were identified, ST3531, ST3540, ST3562 and ST3534. Clonal complex (CC) 1 (including the four new ST), known as an epidemic clone, was the dominant CC. However, there were no indications of persistence of particular ST. The resistance toward 11 antibiotic compounds was assessed. Twelve profiles were generated with 75.8% of strains being sensitive to all antibiotic classes and no Methicillin-resistant *S. aureus* (MRSA) strains were found. The enterotoxin-encoding genes involved in food-poisoning, e.g., *sea, sed, see*, and *seg* were targeted by PCR. The two toxin-encoding genes, *sed* and *see*, were not detected. Only three strains (4.5%) harbored *seg* and two of these also harbored *sea*. Despite the isolates being Methicillin-sensitive *S. aureus* (MSSA), the presence of CC1 clones in the processing environment, including some harboring enterotoxin encoding genes, is of concern and hygiene must have high priority to reduce contamination.

## Introduction

*Staphylococcus aureus* is an important opportunistic pathogen that can cause infections in warm-blooded animals and is a leading cause of nosocomial infections in humans and bovine mastitis (Peton and Le Loir, 2014). *S. aureus* is also one of the most common causative agents of food-poisoning (Balaban and Rasooly, 2000; Le Loir et al., 2003; Hennekinne et al., 2012; Kadariya et al., 2014) leading to 241,000 illnesses per year in the United States (Scallan et al., 2011) and is one of the main agents implicated in foodborne diseases in Brazil (Gomes et al., 2013). In foods, enterotoxigenic strains of *S. aureus* can produce heat-stable and protease resistant staphylococcal enterotoxins (SE) causing one of the most common foodborne intoxications (Balaban and Rasooly, 2000; Dinges et al., 2000; Le Loir et al., 2003; Argudín et al., 2010) leading to rapid clinical symptoms such as abdominal cramps, nausea, emesis, and diarrhea (Le Loir et al., 2003; Murray, 2005). More than 20 distinct SE have been described to date including SEA and SED (Jones et al., 2002; Asao et al., 2003; Kérouanton et al., 2007; Pinchuk et al., 2010; Sato’o et al., 2014; Solano et al., 2014; Arfatahery et al., 2016) responsible for the majority of staphylococcal food-poisoning (SFP), and also in fewer cases SEB, SEC, SEE, SEG, SEH, and SEI (Chen et al., 2004; Ikeda et al., 2005; Ostyn et al., 2010; Pinchuk et al., 2010). In 2012, 346 outbreaks caused by SE were reported in Europe (EFSA and ECDC, 2014). However, not every *S. aureus* strains carry SE-encoding genes (Lindberg et al., 2000; Le Loir et al., 2003) and the risk for human beings from different *S. aureus* may thus vary.

*Staphylococcus aureus* has been isolated from several foods including several ready-to-eat products (Asao et al., 2003; van Loo et al., 2007; Schmid et al., 2009; Ostyn et al., 2010; Rizek et al., 2011; Baumgartner et al., 2014; Hao et al., 2015) and improper staff hygiene as well as poor surface sanitation are some of the main causes of cross-contamination (Hatakka et al., 2000; Asao et al., 2003; Lues and Van Tonder, 2007; André et al., 2008). *S. aureus* is capable of surviving on dry stainless steel and it can easily be transferred from sponges to stainless steel surfaces and subsequently to food products (Kusumaningrum et al., 2003). In the dairy industry, *S. aureus* can be introduced at almost every step of the production. Beside handlers and environment, the raw milk can be one route of introduction as a consequence of subclinical or clinical bovine mastitis. *S. aureus* can be shed into the milk (Kérouanton et al., 2007; Peton and Le Loir, 2014) and produce SE which represents one of the most common food safety concerns from raw milk products (Fagundes et al., 2010; Silveira-Filho et al., 2014). In addition, SE can be produced in the product during storage if conditions allow growth of *S. aureus*.

Dairy production is an important industry worldwide. In Brazil, up to 30% of the milk production comes from small producers (Lee et al., 2012) and soft cheeses such as Minas frescal type are widely consumed. This particular cheese is non-ripened semi-fat and high moisture (Saboya et al., 1998) which offers suitable growth conditions for many pathogens including *S. aureus* that has frequently been the cause of foodborne diseases (Veras et al., 2008). In 1996, the Brazilian Ministry of Agriculture (MAPA) implemented microbiological regulations for cheeses, subsequently extending specific maximum *S. aureus* contamination levels (e.g., 10^3^ CFU/g for coagulase positive staphylococci) for Minas frescal cheese (Ministério da Agricultura Brasil, 1997). However, our knowledge regarding *S. aureus* occurrence in Brazilian dairy plants is still limited.

Besides food-poisoning, *S. aureus* is a major concern due to its ability to acquire antibiotic resistance genes and to disseminate and to withstand treatment (Khan et al., 2000a,b; Holden et al., 2004; de Lencastre et al., 2007; Pesavento et al., 2007). Methicillin-resistant *Staphylococcus aureus* (MRSA) strains are responsible for the majority of nosocomial infection worldwide (ECDC, 2014; EFSA and ECDC, 2014) and there is an increase in MRSA detection in the community, livestock environment and food (Normanno et al., 2007; van Loo et al., 2007; EFSA and ECDC, 2014). Thus, despite being mostly problematic in infection, it raises concern that foods could potentially also be a vehicle of antibiotic resistant *S. aureus* strains. Epidemiological studies have been used to track the contamination origin and the diversity of clinical isolates for many years. However, particular focus has been on MRSA isolates (Chambers and Deleo, 2009). Randomly amplified polymorphic DNA (RAPD) has been used to determine the relatedness of *S. aureus* from cattle, pigs, chickens, and humans (Lee, 2003) concluding that humans can acquire MRSA infections from livestock *via* contaminated food products. This reinforces that contamination of dairy processing environment, milk and dairy products by *S. aureus* is a public health concern (Fagundes et al., 2010; Oliveira et al., 2011). However, few studies have assessed the global epidemiology of *S. aureus* of animal origin and their dissemination into the food chain (Lee et al., 2012; Sobral et al., 2012; Lima et al., 2013). Different from RAPD and among the various molecular typing methods, multi-locus sequence typing (MLST) (Enright et al., 2000) provides global epidemiology data. Indeed, MLST can be used to track contamination sources, to indicate persistence of particular clones and to identify epidemic clones. However, studies investigating the molecular characteristic of *S. aureus* from dairy processing environment and dairy products in Brazil are scarce.

The objectives of this study were: (*i*) to estimate the incidence of *S. aureus* contamination in the Brazilian dairy industry of the Southeast and Midwest regions, (*ii*) to determine the genetic diversity of the isolates by MLST as well as to evaluate their reoccurrence and eventually persistence, and (*iii*) to assess some of the virulence factors involved in health risk such as antibiotic resistance spreading by determining the antibiotic profile, the hemolytic trend potential and to investigate the possible food-poisoning ability of the isolates by detecting the presence of enterotoxin encoding genes.

## Materials and Methods

### Bacterial Strains and Growth Media

*Staphylococcus aureus* isolates collected from Brazilian dairies were investigated in this study (Supplementary Table 1) and sampled as described in Section “Sampling Procedure and Detection of Putative *S. aureus*.” Isolation and phenotypic tests were performed using Brain–Heart Infusion (BHI) broth (Oxoid, United Kingdom), BHI agar [BHI broth, 1.5% agar (AppliChem, Germany)], Tryptic Soy Broth [TSB (Oxoid, United Kingdom)], Tryptic Soy Agar [TSA (Oxoid, United Kingdom)] supplemented with 0.6% yeast extract (TSAYE), DNase Test Agar with Methyl Green (Difco, Detroit, MI, United States), Mueller Hinton II Agar [MHA (BD Diagnostics, Franklin Lakes, NJ, United States)], Mannitol Salt Agar (MSA) and Baird Parker agar (Oxoid, United Kingdom) supplemented with egg-yolk emulsion (BP). Blood agar was prepared by supplementing TSA with defibrinated sheep blood (Statens Serum Institut, Denmark) at 5% (vol/vol). Unless otherwise specified, isolates were grown at 37°C and liquid cultures were incubated under shaking conditions at 250 rpm. The isolates were stored in BHI containing 20% (vol/vol) glycerol (Merck, Germany) at -80°C.

### Processing Plants and Sample Collection

Five dairy processing plants in São Paulo state and Minas Gerais state (Southeast region) and Goiás state (Midwest region) in Brazil were sampled over an 8 months period from December 2013 to July 2014. In São Paulo state, two dairy plants were evaluated, one small-scale with a volume of 7,000 L cow’s milk processed per day from 60 producing farms, and the other of medium-scale that processed nearly 60,000 L per day collected from 150 farms. Two out of the three sampled dairies in Goiás state were small-scale dairies, processing about 3,000 L per day provided by around 30 farms. The third dairy was an artisanal cheese manufacturing plant, producing about 50 cheeses per day, using 250 L per day, supplied by just one cattle herd, raised on site where the cheese factory is located and using unpasteurized milk. Four categories of samples were investigated: raw material (bulked raw milk, pasteurized milk, brine, curd), food-contact surfaces (milk filter, vats, spatulas, paddle, knife, tank, picking machine strainer, hand handler, worker gloves, cheese box, shelf, gallon of milk, cloth mold, mold, plastic crates, curd tube, tables), non-food contact surfaces (pallet, wall, floor, drain, water, cleaning brush, sink, tables, gloves, and staff’s boot), and final product (cheese surfaces, cheese) as shown in **Table 1** and Supplementary Table 1. The five dairies followed the same cheese processing procedure using milk and no lactic acid bacteria in the production. The milk was pasteurized except in one dairy where they used unpasteurized milk and it was chosen to represent this type of Brazilian dairy (corresponding to 20–40% of dairies in this country).

**Table 1:** Distribution of samples collected in this study in five dairies between December 2013 and July 2014 in the states of São Paulo, Minas Gerais and Goiás (Brazil).

Dairy	Sampling date	Number of samples per category				Total
		Raw material	Product	Food contact surfaces	Non-food contact surfaces	
**Goiás state**						
1	December 2013	4	3	9	9	25
2	February 2014	4	9	12	5	30
3	March 2014	4	7	13	6	30
	July 2014	4	9	11	6	30
**Minas Gerais state**						
4	December 2013	3	5	9	4	21
	January 2014	2	7	8	6	23
	February 2014	3	21	21	8	53
	March 2014	3	9	17	14	43
	July 2014	3	4	11	10	28
**São Paulo state**						
5	December 2013	4	11	10	5	30
	January 2014	3	7	7	7	24
	February 2014	3	9	8	4	24
	March 2014	3	13	15	4	35
	July 2014	3	4	10	8	25
Total		46	118	161	96	421

*The samples are grouped according to the sample category, e.g., raw material, product, food-contact surfaces, and non-food-contact surfaces depending on the date and the sample category.*

### Sampling Procedure and Detection of Putative *S. aureus*

A total of 421 samples were collected at different stages of processing following the method described by Oxaran et al. (2017). Briefly, raw material (*n* = 46), food-contact surfaces (*n* = 161), non-food-contact surfaces (*n* = 96), and products (*n* = 118) (**Table 1** and Supplementary Table 1) were sampled using swab or sponge or by taking 25 g of cheese. Samples were transferred to the laboratory in cooling boxes containing ice packs. Homogenized samples in PSW were diluted 10-fold and 0.1 mL plated on BP to isolate putative *S. aureus*. After 24–48 h incubation at 35°C, up to three characteristic colonies of *S. aureus* (typical black, convex colonies, and with halo) were purified on TSAYE and incubated at 35°C for 24–48 h. Putative *S. aureus* isolates were stored for further analysis.

### Phenotypic Tests

Shape and motility was determined by microscopy using an Olympus microscope (BX51). Gram-reaction was assessed by the 3% KOH method (Halebian et al., 1981). Catalase was tested using 3% hydrogen peroxide (Merck, Germany). Mannose fermentation and tolerance to high salt concentration was assessed on MSA incubated at 35°C for 48 h (Chapman, 1945). DNase activity was tested on DNase Test Agar with Methyl Green incubated at 35°C for 48 h. The production of free coagulase (Essers and Radebold, 1980) was tested using rabbit plasma (BioRad, France) and the presence of clumping factor as well as Protein A were tested using the Pastorex Staph-Plus Kit (BioRad, France) according to the manufacturer’s recommendations.

### DNA Manipulation

Genomic DNA was extracted using the Dynabeads^®^ DNA DIRECT Universal kit (Invitrogen) on a 24 h culture. For each PCR reaction, 1 μL of extracted genomic DNA was used with primers listed in **Table 2**. PCR for the species identification and MLST were performed using the TEMPase Hot Start 2X Master Mix Blue II (Ampliqon, Denmark) and the Maxima Hot Start Taq Polymerase (Fermentas, Waltham, MA, United States) was used for toxin-encoding gene amplification, both were performed in a 25 μL reaction following the manufacturer’s instruction. Reactions were done in a Veriti Thermal Cycler (Applied Biosystems, 96 Well Model 9902). PCR products were purified using 10 U Exonuclease I (Thermo Fisher Scientific, Waltham, MA, United States) and 0.5 U Fast Alkaline Phosphatase (Thermo Fisher Scientific, Waltham, MA, United States) and incubated at 37°C for 15 min followed by an inactivation step at 85°C for 15 min (Werle et al., 1994). Macrogen Europe (The Netherlands) sequenced the gene fragments using the same primer used for the PCR amplification.

**Table 2:** List of primers used for the identification of *Staphylococcus aureus* based on 16 rRNA gene locus sequencing and for detection of four toxin encoding genes involved in food-poisoning, e.g., *sea, sed, see*, and *seg.*

Gene	Primer	Sequence (5′–3′)	Reference
**Species identification**			
*16S rRNA*	27 F	AGA GTT TGA TCM TGG CTC AG	Lane, 1991
	1492 R	TAC GGY TAC CTT GTT ACG ACT T	Turner et al., 1999
**Toxin**			
*sea*	SA-U	TGTATGTATGGAGGTGTAAC	Sharma et al., 2000
	SA-A	ATTAACCGAAGGTTCTGT	Sharma et al., 2000
*sed*	SED-1	GAATTAAGTAGTACCGCGCTAAATAATATG	Jarraud et al., 2002
	SED-2	GCTGTATTTTTCCTCCGAGAGT	Jarraud et al., 2002
*see*	SEE-1	CAAAGAAATGCTTTAAGCAATCTTAGGC	Jarraud et al., 2002
	SEE-2	CACCTTACCGCCAAAGCTG	Jarraud et al., 2002
*seg*	SEG-1	AATTATGTGAATGCTCAACCCGATC	Jarraud et al., 2002
	SEG-2	AAACTTATATGGAACAAAAGGTACTAGTTC	Jarraud et al., 2002

### *S. aureus* Species Confirmation

The *S. aureus* identification was confirmed by 16S rRNA gene sequencing using primers 27F (Lane, 1991) and 1492R (Turner et al., 1999) (**Table 2**). The consensus sequence for each 16S rRNA locus was analyzed using the BLASTn algorithm on National Center for Biotechnology^1^.

### Multi-locus Sequence Typing

The sequence type (ST) of each *S. aureus* isolate was determined by the method described by Enright et al. (2000). The internal fragment of seven housekeeping genes *arcC* (Carbamate kinase), *aroE* (shikimate dehydrogenase), *glpF* (glycerol kinase), *gmk* (guanylate kinase), *pta* (phosphate acetyltransferase), *tpi* (triosephosphate isomerase), *yqiL* (acetyle coenzyme A acetyltransferase), was amplified by PCR following the guideline on http://pubmlst.org/saureus/. The sequences were processed using CLC Main Workbench 7 (CLC Bio-Qiagen, Denmark) and analyzed using the *S. aureus* MLST database^2^ to assign the allele type and thus the ST. The profiles obtained including the new alleles and new profiles were submitted to the MLST database to contribute to the resource for *S. aureus* global epidemiology.

### Virulence Factor Phenotypes

#### Hemolytic Activity

Hemolytic activity was tested on blood agar incubated at 35°C for 48 h and classified as α-hemolytic (partial hemolysis), β-hemolytic (complete hemolysis), α′-hemolytic (weak partial hemolysis) or γ-hemolytic (non-hemolytic bacteria).

#### Antibiotic Susceptibility

Overnight cultures were adjusted to 0.5 McFarland (Biomerieux, France) suspensions (corresponding to 1–3 × 10^8^ CFU/mL) and were inoculated by swabbing onto MHA in 120 mm × 120 mm Petri dishes (Almeco, Denmark). Eleven antibiotic disks (Oxoid, United Kingdom), Penicillin G (PEN, 10 U), Erythromycin (ERM, 15 μg), Tetracycline (TET, 30 μg), Clindamycin (CLIN, 2 μg), Gentamicin (GEN, 10 μg), Ciprofloxacin (CIP, 5 μg), Vancomycin (VAN, 5 μg), Trimethoprim-Sulfamethoxazole (SXT, 25 μg), Chloramphenicol (CAM, 30 μg), Cefoxitin (CEF, 30 μg), Bacitracin (BAC, 10 U) were tested. The diameter of inhibition zones was determined after 16–20 h and after 24 h of incubation at 35°C. The interpretation of the inhibition zone diameters was based on the EUCAST values (EUCAST, 2015) except for bacitracin and vancomycin inhibition zones which were compared to the Food and Drug Administration guidelines (FDA, 2006, 2009). *S. aureus* ATCC 25923 was used as control strain.

#### Toxin-Encoding Gene Detection

The presence of the genes encoding four toxins found in SFP was determined by PCR using the primers listed in **Table 2**. The most two common toxin encoding genes involved in SFP, *sea* and *sed*, and also the not so common SFP toxin encoding genes, *see* and *seg*, were targeted. The *S. aureus* strains ATCC 29213 (*sea, seg*), DSM 18588 (*sed*), DSM 18589 (*see*) were used as positive controls.

## Results

### *S. aureus* Is a Contaminant of the Brazilian Dairy Industry

Thirty-one of 421 samples were positive for *S. aureus* (Supplementary Table 1). Strains were isolated from between 3 and 5 colonies from each sample showing typical *S. aureus* colony macroscopy on Baird Parker plates. A total of 66 isolates were identified as *S. aureus* being Gram positive, cocci shaped cells, catalase positive, DNase positive, growing in presence of high salt concentration, fermenting mannitol, as well as producing free and bound coagulase. The 16S rRNA gene sequencing confirmed the *S. aureus* identification for all 66 isolates. Four out of five dairies were positive for *S. aureus*, leading to an overall contamination rate of 7.4% (*n* = 31). The average contamination rate varied among dairies from 0% (*n* = 0) in Dairy 1 to 63.3% (*n* = 19) in Dairy 2 (**Table 3**). The dairies 3, 4 and 5, had contamination rates of 8.3% (*n* = 5), 1.2% (*n* = 2), and 3.6% (*n* = 5), respectively. Based on the overall contamination rate of *S. aureus* positive samples (*n* = 31), *S. aureus* was isolated in all four sampling categories (**Table 3**): raw material (10.9%), product (7.6%), food-contact surfaces (8.1%), and non-food-contact surfaces (4.2%).

**Table 3:** Overall occurrence of *S. aureus* per dairy in the five dairies investigated grouped per sampling point category, e.g., raw material, product, food-contact surfaces, and non-food-contact surfaces.

	Number of positive samples/total number of samples (%)				
Dairy	Raw material	Product	Food-contact surface	Non-food-contact surface	Total
1	0/4	0/3	0/9	0/9	0/25 (0)
2	2/4	8/9	6/12	3/5	19/30 (63.3)
3	3/8	0/16	1/24	1/12	5/60 (8.3)
4	0/14	0/46	2/66	0/42	2/168 (1.2)
5	0/16	1/44	4/50	0/28	5/138 (3.6)
Total	5/46 (10.9)	9/118 (7.6)	13/161 (8.1)	4/96 (4.2)	31/421 (7.4)

### Prevalence of CC1 *S. aureus* Isolates in the Brazilian Dairy Industry

Using MLST, the 66 *S. aureus* isolates were separated into 11 ST which were grouped into six clonal complexes (CCs) (**Table 4**). Of the 66 isolates, only 19 could be assigned to a known ST: ST1 (*n* = 7), ST188 (*n* = 3), ST97 (*n* = 3), ST398 (*n* = 2), ST5 (*n* = 1), ST30 (*n* = 2), and ST126 (*n* = 1). Besides the seven known ST, four new ST were found according to the MLST database^2^. The four new STs were assigned to 47 of the isolates. Due to a single point mutation, the new ST3531 (*n* = 41), ST3534 (*n* = 1), and ST3540 (*n* = 2) harbored a new allele type of the *yqiL* locus, 508, *gmk* locus, 291, or *glpF* locus, 515, respectively. All new allele types and thus ST were recorded on the MLST database. All of the seven allele types of the new ST, ST3562 (*n* = 3), were already assigned but the concatenation was novel and the isolate profile was submitted to the MLST database.

**Table 4:** MLST scheme of the *S. aureus* isolates from dairies in the Southeast and Midwest regions of Brazil.

ST	Allele types							CC	Number of isolates	Dairy
	*arcC*	*aroE*	*glpF*	*gmk*	*pta*	*tpi*	*yqiL*			
1	1	1	1	1	1	1	1	1	7	3, 5
188	3	1	1	8	1	1	1	1	3	4
3531	1	1	1	1	1	1	508	1	41	2
3534	3	1	1	291	1	1	1	1	1	4
3540	1	1	515	1	1	1	1	1	2	2
3562	1	1	211	1	1	1	1	1	3	3
5	1	4	1	4	12	1	10	5	1	3
30	2	2	2	2	6	3	2	30	2	5
97	3	1	1	1	1	5	3	97	3	3, 5
126	3	68	1	4	1	5	40	126	1	3
398	3	35	19	2	20	26	39	398	2	2, 4

*Allele types of each locus, sequence types (ST) and clonal complex (CC) for the 66 *S. aureus* isolates sampled from the dairies are listed. For each ST, the number of isolates and the dairies are indicated.*

### Heterogeneous Distribution of ST among the Dairies

ST3531 (*n* = 41) and ST3540 (*n* = 2) were only found in Dairy 2 with ST3531 being the predominant type (*n* = 41 out of 44 positive isolates). In this dairy, ST398 (*n* = 1) was also found. The ST3562 (*n* = 3) as well as ST5 (*n* = 1) and ST126 (*n* = 1) were specific to Dairy 3 and ST1 (*n* = 4) and ST97 (*n* = 2) were also identified. ST188 (*n* = 3), ST3534 (*n* = 1), and ST398 (*n* = 1) were isolated in Dairy 4. Dairy 5 was contaminated with ST1 (*n* = 3), ST30 (*n* = 2), and ST97 (*n* = 1).

### Does *S. aureus* Reoccur and Persist in the Brazilian Dairy Industry?

The assigned ST were compared to the sampling points in each dairy and there was no correlation between particular ST at one specific sampling point. However, ST3531 was found at many different sampling points (wall, curd, cloth mold, cheese surface, sink, handler’s hand, floor, brine, processing tank, cheese, and paddle) in Dairy 2 (Supplementary Table 2). ST1 was found at two different sampling points both in Dairy 3 (curd and unpasteurized milk) and in Dairy 5 (bucket and cold chamber shelf). The results also showed that one sampling point, the unpasteurized milk (Dairy 3) was contaminated with *S. aureus* of three different ST (ST1, ST126, ST97). The ST were compared to the sampling time points in each dairy. Only ST1 and ST30 in Dairy 3 and 5, respectively, were occurring at two different time points indicating a re-introduction of these ST.

### Virulence Related Phenotypes

#### Hemolytic Activity

Out of the 66 isolates, 29 lysed the red blood cells completely and were β-hemolytic. Thirteen strains were α-hemolytic. Twenty-three isolates produced a weak α′-hemolytic zone and one isolate (Sa8) did not display hemolytic activity, being γ-hemolytic (Supplementary Table 2).

#### Antibiotic Susceptibility

The 66 isolates were all susceptible to four antibiotics: trimethoprim-sulfamethoxazole, chloramphenicol, cefoxitin, and bacitracin (**Table 5**). Resistance toward one or more antibiotics was observed for 24.2% of the isolates (*n* = 16). Seven isolates were only resistant to one antibiotic being either ciprofloxacin (1.5%, *n* = 1) or penicillin G (9.1%, *n* = 6). Combination of resistance toward two to four antibiotics was observed for nine isolates (13.6%) (**Table 5**). None of the 66 isolates were methicillin resistant. No isolates were resistant to vancomycin but 21 isolates showed an intermediate tolerance to this antibiotic. One of the vancomycin intermediate tolerant isolates (Sa44) was also intermediate tolerant to tetracycline (Supplementary Table 2).

**Table 5:** Number of isolates per antibiotic susceptibility profile related to the sequence type (ST).

Number of isolates	Antibiotic susceptibility								ST
	PEN	ERM	TET	CLIN	GEN	CIP	VAN	SXT, CAM, CEF, BAC	
36	S	S	S	S	S	S	S	S	3531,3540, 3562,126, 1
14	S	S	S	S	S	S	I	S	1, 3531,3562
3	R	S	S	S	S	S	S	S	5,3531,30
2	R	S	S	S	S	S	I	S	30, 97
1	S	S	S	S	S	R	I	S	3531
1	R	S	I	S	S	S	I	S	3531
1	S	S	R	S	R	S	S	S	1
2	R	R	S	S	S	S	S	S	398
2	R	S	R	S	S	S	S	S	188
1	R	R	S	R	S	S	I	S	97
1	R	R	R	R	S	S	S	S	97
2	R	R	R	R	S	S	I	S	188,3534

*Sensitivity (S), intermediate tolerance (I), and resistance (R) was determined for all 11 antibiotics tested: Penicillin G (PEN), Erythromycin (ERM), Tetracycline (TET), Clindamycin (CLIN), Gentamicin (GEN), Ciprofloxacin (CIP), Vancomycin (VAN), Trimethoprim-Sulfamethoxazole (SXT), Chloramphenicol (CAM), Cefoxitin (CEF), and Bacitracin (BAC).*

#### Detection of Food-Poisoning Related Toxin-Encoding Genes

Using PCR, none of the isolates was positive for detection of gene encoding toxins D and E, e.g., *sed* and *see* respectively. Three isolates out of 66 were positive for *seg*, including two isolates harboring both genes *seg* and *sea* (Supplementary Table 2).

## Discussion

Minas frescal cheese is a widely consumed soft cheese in Brazil and it has been associated with *S. aureus* food-poisoning (de Almeida Filho and Nader Filho, 2000; Araújo et al., 2002). Most of the dairies investigated in the present study were positive for *S. aureus*. This is in concordance with the general trend that this pathogen is a common contaminant of dairy industries worldwide, including Brazil (de Almeida Filho and Nader Filho, 2000; Araújo et al., 2002; Carvalho et al., 2007; André et al., 2008; Fagundes et al., 2010) where contamination rates can reach up to 77.0% (Araújo et al., 2002; André et al., 2008; Moraes et al., 2009). Here, we identified one dairy with a high level of contamination (63.3%, Dairy 2). The four other dairies had a lower contamination rate (from 0 to 8.3%) which is similar to the rate described by Lee et al. (2012) but lower than generally reported in the literature (Araújo et al., 2002). *S. aureus* was identified in all four sampling categories (raw material, product, food-contact surfaces, and non-food-contact surfaces) but the contamination rates varied. The highest overall contamination rate was observed for the raw material category (10.9%), however, only two out of the five dairies were positive for *S. aureus* presence in this category. This included *S. aureus* isolation from the milk in the only dairy producing cheese from unpasteurized milk (Dairy 3), however this type of dairy did not show the highest contamination rate of any of the other sampling categories. Contamination of raw milk by *S. aureus* has previously been reported and linked to milk from animals with mastitis (Jørgensen et al., 2005; da Costa Krewer et al., 2015). The frequency of *S. aureus* contamination of dairy cows is quite high in Brazil (da Costa Krewer et al., 2015) and this could also be one of the contamination sources for Dairy 3. However, this is likely not the only pathogen introduction route for all of the investigated dairies. The overall contamination rates of the food-contact surfaces and products were slightly lower than that of the raw material. Nevertheless, at least one food-contact surface sample was positive for *S. aureus* in all of the dairies where the pathogen was detected. *S. aureus* was also isolated from the handler’s hands, gloves, and utensils in three of the dairies (Dairy 2, 4, and 5). Several studies have reported that contamination by *S. aureus* is mainly due to transfer between handlers and the equipment thus incriminating the lack of good manufacturing practices (GMPs) (Lee et al., 2012; Kümmel et al., 2016). Altogether, it hints towards a human spread of the pathogen in the investigated dairies and a need for improvement in the hygienic practices. However, further monitoring is needed to confirm this speculation.

We typed the *S. aureus* isolates choosing MLST among the different epidemiological methods that have previously been applied to study the relatedness of *S. aureus* isolates in food production plants (Tondo et al., 2000; Lee, 2003; Kérouanton et al., 2007; Rabello et al., 2007; Agersø et al., 2012; Lee et al., 2012; Yan et al., 2012; Shepheard et al., 2013; Rešková et al., 2014; McMillan et al., 2016). Indeed, MLST provide data that can be compared on a global scale and allow typing of important *S. aureus* clones such as ST398 involved in human and animal infections that are non-typable by the standard PFGE method (using *sma*I) due to DNA methylation (Bens et al., 2006). The 66 isolates identified in this study belonged to six different CC and to seven known ST (ST1, ST5, ST30, ST97, ST126, ST188, and ST398) and four new ST (ST3531, ST3534, ST3540, ST3562). According to the MLST database^2^ and previous studies on milk and dairy products, all of the known ST have previously been isolated in Brazil (Vivoni et al., 2006; Rabello et al., 2007; Silva et al., 2013, 2014). In three instances, more than one ST was identified from one single sample, e.g., ST3531 and ST3540 being identified from the same cheese surface sample (Supplementary Table 2), which shows that more than one *S. aureus* clone were present and thereby supports the requirement of analyzing more than one colony per sample (Mongkolrattanothai et al., 2011; Votintseva et al., 2014). We did not find any indications of persistence as it has been seen in other studies (Koreňová et al., 2015) but our results indicate that *S. aureus* was continuously reintroduced into the dairies.

The majority of the isolates (44 out of 66) including the four novel ST belonged to CC1. Although several other studies have found large diversity regarding *S. aureus* contamination (Oliveira et al., 2016), Silva et al. (2013) also found that CC1 (ST1) was the main contaminant of milk from Brazilian cows infected with mastitis due to MSSA and also identified a new ST belonging to CC1. So far, the clinically related data represents the largest amount of available data on *S. aureus* epidemiology in Brazil and using other molecular typing methods, it has been shown that the genetic diversity of this pathogen in Brazilian hospitals is limited (Teixeira et al., 1995) and thus is in concordance with our results observing a low genetic diversity as well as the predominance of the CC1. All together it reinforces that clinical clones are now spreading into other niches and potentially into the food industry (Pesavento et al., 2007; Choudhury et al., 2012). In addition, isolates from different sampling points in one dairy (Dairy 2) belonged to the same ST3531, being *per se* epidemiologically related and thus suggesting a potential dissemination of one clone in the dairy. Investigating *S. aureus* from milk and the milking environment in São Paulo by PFGE (Lee et al., 2012), reached the same conclusion. To a lesser extent, the other ST belonging to different CC observed in this study have been sporadically reported in bovine dairy or products. Of concern, ST398 has previously been described as a human public health threat in Europe due to its ability to disseminate in humans and pigs (Armand-Lefevre et al., 2005; Voss et al., 2005; Price et al., 2012) as well as its potential antibiotic resistance pattern. In Brazil, MRSA ST398 has been reported in bovine mastitis (Silva et al., 2014). The two ST398 isolates found in this study were MSSA, and in China, MSSA ST398 has also been found in food products (Yan et al., 2014; Li et al., 2015b). Although the main pathogenicity determinants of MSSA ST398 are still unknown, the presence of these isolates along the food processing chain is of concern for human health. Indeed, MSSA ST398 has recently been involved in human infections (Valentin-Domelier et al., 2011; Mediavilla et al., 2012; David et al., 2013). In previous reports from Brazilian dairies, ST1, ST97, and ST126 were commonly identified (Rabello et al., 2007; Silva et al., 2013; Oliveira et al., 2016) and ST97 has also been found in dairies worldwide (Smith et al., 2005). ST126 that belongs to CC126 is usually associated with ruminants but not humans (Smith et al., 2005; Aires-de-Sousa et al., 2007; Rabello et al., 2007), hence, suggesting a bovine origin for those isolates. The ST5/CC5 was originally described in poultry infections but it can also be found among human isolates (Lowder et al., 2009). Moreover, ST5 was also present in dairy environments in the US (Matyi et al., 2013) and in the Brazilian dairy industry (Silva et al., 2013). Highlighted by those results, ST found in the dairy plants investigated here belonged to ST or CC previously involved into food-poisoning (ST1, ST5, ST30, and ST188) worldwide (Cha et al., 2006; Yan et al., 2012; Li et al., 2015a).

Le Loir et al. (2003) have reported that 25% of *S. aureus* strains isolated from food are enterotoxigenic and staphylococcal enterotoxins are responsible for food-poisoning outbreaks worldwide including from dairy products (Do Carmo et al., 2004; Loncarevic et al., 2005). Here, a small number of strains (4.5%) harbored SE-encoding genes involved in SFP. The number of strains harboring SE-encoding genes varies, especially depending on the geographical area (Jørgensen et al., 2005; Neder et al., 2011; Riva et al., 2015) and has ranged from 0 to 93.6% (Do Carmo et al., 2004; Fagundes et al., 2010; Rall et al., 2014; Silveira-Filho et al., 2014). SEA is the most prevalent toxin involved in SFP worldwide and *sea* as well as *seb* were the predominant SE-encoding genes found to cause SFP in Brazil (Veras et al., 2008). Here, only two isolates harbored *sea*, which is less frequent than previously reported. The second SE-encoding gene found in three isolates was *seg*. The *seg*/*sei* genes were recently reported to be the most prevalent among mastitis milking cows and cheese manufacturing in Brazil (Arcuri et al., 2010; Silveira-Filho et al., 2014). Linking ST to toxin gene presence, only ST5 and ST30 strains harbored SE-encoding genes in this study. In fact, assessing the MLST profile to the toxigenic potential of *S. aureus* food sources in China (Chang et al., 2016) has illustrated the specific ability of ST5 strains to harbor and to produce SE while the other ST, including ST1, ST188, ST97 and ST398, did not, as we also noticed.

*Staphylococcus aureus* is known for its ability to gain antimicrobial resistance and attention has been given to multi-drug as well as methicillin resistant strains and in particular to highly virulent MRSA strains. Several studies have shown that about 20% of the isolates were MRSA (Türkyilmaz et al., 2010; Riva et al., 2015), with in particular in Brazil methicillin resistance occurrence ranges from 5.5 to 37.2% in milk, dairy processing environment and dairy products (André et al., 2008; Silveira-Filho et al., 2014; Oliveira et al., 2016). However in this study, none of the isolates were classified as MRSA which corroborates with one previous study (Silveira-Filho et al., 2014). Additionally, no isolates were resistant toward vancomycin concurring with previous reports in the Brazilian dairy industry (André et al., 2008; Silveira-Filho et al., 2014; Oliveira et al., 2016). Hence, there are yet no indications of any multi-drug resistant MRSA emerging in the Brazilian dairy industry. The highest resistance rates were observed for penicillin G (20.9%), erythromycin (9.0%), and tetracycline (9.0%) similar to findings by Oliveira et al. (2016). Resistance to penicillin is frequently found in dairy herds worldwide, the occurrence rates ranging from 10 to 70% of the isolates being resistant with in particular 47.6% in Uruguay and Argentina (Calvinho et al., 2002; Gianneechini et al., 2002) as well as 69.9% in Brazil (André et al., 2008). However, resistance to the widespread administration of these specific antibiotics to control and treat infections in dairy farms including mammary infections reached lower value here. Thus, in this specific region the use of β-lactams as antibiotic treatment may have only triggered the increase of resistance occurrence to β-lactams such as penicillin. Moreover, the co-resistance pattern of tetracycline and macrolide (erythromycin) has frequently been detected in ST1 (MRSA) in Italy (Battisti et al., 2010; Franco et al., 2011; Carfora et al., 2015) which corroborates with our data. Clindamycin, gentamicin, and ciprofloxacin are commonly used in Brazil to treat bovine mastitis and it has been suggested that these antibiotics trigger a selective pressure in dairy farms. Indeed, all of the isolates from dairy farms in Northeastern Brazil collected by Silveira-Filho et al. (2014) were resistant toward gentamicin and bacitracin. However, the resistance observed toward these antibiotics was very limited here as also observed by others (André et al., 2008). Nevertheless, the resistance to more than one antibiotic of some isolates, observed here, could pave the way of the resistant determinant dissemination.

## Conclusion

Some Brazilian dairy processing plants and cheese products have frequent occurrence of *S. aureus*. The genetic diversity is low and could indicate a few sources of contamination and, if identified could form the basis for an intervention strategy.

## Author Contributions

Study concept and design: VO and LG. Planning and sampling at dairies: LC, SL, CC, EPDM, VA, and CFdO. Analysis and interpretation of data: KD, SL, LC, and VO. Drafting of the manuscript: VO. Critical revision of the manuscript: LG, KD, EPDM, VA, CC, VO, and CFdO.

## Conflict of Interest Statement

The authors declare that the research was conducted in the absence of any commercial or financial relationships that could be construed as a potential conflict of interest.

## Footnotes

1 http://blast.ncbi.nlm.nih.gov/Blast.cgi

2 http://pubmlst.org/saureus/
