# Supplementary material for: Staphylococcus aureus in Some Brazilian Dairy Industries: Changes of Contamination and Diversity
Source: Front Microbiol. 2017 Oct 24;8:2049. doi: 10.3389/fmicb.2017.02049 (PMC5662873; doi:10.3389/fmicb.2017.02049)
Supplement: Supplementary file 1 [file Table_1.DOCX]

Supplementary Material

*Staphylococcus aureus* in some Brazilian dairy industries: changes of contamination and diversity

Karen Kiesbye Dittmann, Luíza Toubas Chaul, Sarah Hwa In Lee, Carlos Humberto Corassin, Carlos Augusto Fernandes de Oliveira, Elaine Cristina Pereira De Martinis, Virgínia Farias Alves, Lone Gram*, Virginie Oxaran

*** Correspondence:** Lone Gram: [gram@bio.dtu.dk](mailto:gram@bio.dtu.dk)

## Supplementary Table 1

Sampling plan for five Brazilian dairies between December 2013 and July 2014. Description of each sampling point, date and dairy and indication of number of samples positive for *S. aureus* and number of positive *S. aureus* isolates. Each positive isolate was analyzed as described in Supplementary Table 2.

| Dairy  Date |  | Total number of samples per sampling | Sampling point | Number of sample per sampling point | Number of positive sample | Number of positive isolate |
| --- | --- | --- | --- | --- | --- | --- |
| 1 | Dec-13 | 25 | Pasteurized milk | 1 | 0 | 0 |
|  |  |  | Unpasteurized milk | 1 | 0 | 0 |
|  |  |  | Brine with cheese | 1 | 0 | 0 |
|  |  |  | Brine without cheese | 1 | 0 | 0 |
|  |  |  | Mozzarella cheese surface (before packing) | 1 | 0 | 0 |
|  |  |  | Mozzarella cheese packed | 1 | 0 | 0 |
|  |  |  | Mozzarella cheese surface (from package) | 1 | 0 | 0 |
|  |  |  | Paddle to mix the pasteurized milk | 1 | 0 | 0 |
|  |  |  | Wall-Cold chamber 3 | 1 | 0 | 0 |
|  |  |  | Wall-Cold chamber 1 | 1 | 0 | 0 |
|  |  |  | Wall-Brining room | 1 | 0 | 0 |
|  |  |  | Knife used to cut the cheese | 1 | 0 | 0 |
|  |  |  | Floor-Cold chamber 1 | 1 | 0 | 0 |
|  |  |  | Floor-Processing room | 1 | 0 | 0 |
|  |  |  | Floor-Brining room | 1 | 0 | 0 |
|  |  |  | Floor-Cold chamber 3 | 1 | 0 | 0 |
|  |  |  | Processing tank | 1 | 0 | 0 |
|  |  |  | Pallet-Cold chamber pallet | 1 | 0 | 0 |
|  |  |  | Picking machine | 1 | 0 | 0 |
|  |  |  | Strainer | 1 | 0 | 0 |
|  |  |  | Groove | 1 | 0 | 0 |
|  |  |  | Handler's hand | 1 | 0 | 0 |
|  |  |  | Cheese box after the press | 1 | 0 | 0 |
|  |  |  | Picking machine | 1 | 0 | 0 |
|  |  |  | Storage shelf | 1 | 0 | 0 |
| 2 | Feb-14 | 30 | Brine | 1 | 1 | 3 |
|  |  |  | Recently prepared Minas cheese surface | 1 | 1 | 4 |
|  |  |  | Refrigerated Minas cheese surface 1 | 1 | 1 | 2 |
|  |  |  | Refrigerated Minas cheese surface 2 | 1 | 0 | 0 |
|  |  |  | Minas cheese surface 1 | 1 | 1 | 2 |
|  |  |  | Minas cheese surface 2 | 1 | 1 | 1 |
|  |  |  | Minas cheese surface 3 | 1 | 1 | 3 |
|  |  |  | Minas cheese 1 | 1 | 1 | 2 |
|  |  |  | Minas cheese 2 | 1 | 1 | 1 |
|  |  |  | Minas cheese 3 | 1 | 1 | 3 |
|  |  |  | Curd | 1 | 0 | 0 |
|  |  |  | Unpasteurized milk | 1 | 0 | 0 |
|  |  |  | Pasteurized milk | 1 | 1 | 1 |
|  |  |  | Clean gallon of milk 1 | 1 | 0 | 0 |
|  |  |  | Clean gallon of milk 2 | 1 | 0 | 0 |
|  |  |  | Cheese surface-Freshly prepared | 1 | 1 | 3 |
|  |  |  | Refrigerated cheese surface | 1 | 0 | 0 |
|  |  |  | Cloth mold | 1 | 1 | 2 |
|  |  |  | Mold for cheese-kept at room temperature | 1 | 0 | 0 |
|  |  |  | Mold for cheese-kept at refrigerated room | 1 | 0 | 0 |
|  |  |  | Paddle to mix unpasteurized milk | 1 | 1 | 5 |
|  |  |  | Wall-Processing room | 1 | 1 | 2 |
|  |  |  | Floor-Processing room | 1 | 1 | 2 |
|  |  |  | Cheese strainer | 1 | 0 | 0 |
|  |  |  | Curd tub | 1 | 1 | 1 |
|  |  |  | Sink near the area of cheese production | 1 | 1 | 2 |
|  |  |  | Processing tank | 1 | 1 | 3 |
|  |  |  | Cleaning brush | 1 | 0 | 0 |
|  |  |  | Handler's hand | 1 | 1 | 2 |
|  |  |  | Drain | 1 | 0 | 0 |
| 3 | Mar-14 | 30 | Unpasteurized milk | 1 | 1 | 4 |
|  |  |  | Pasteurized milk | 1 | 0 | 0 |
|  |  |  | Curd | 1 | 0 | 0 |
|  |  |  | Minas cheese surface 1 | 1 | 0 | 0 |
|  |  |  | Minas cheese 1 | 1 | 0 | 0 |
|  |  |  | Minas cheese surface 2 | 1 | 0 | 0 |
|  |  |  | Minas cheese 2 | 1 | 0 | 0 |
|  |  |  | Minas cheese surface 3 | 1 | 0 | 0 |
|  |  |  | Minas cheese 3 | 1 | 0 | 0 |
|  |  |  | Brine | 1 | 0 | 0 |
|  |  |  | Recently prepared Minas cheese surface | 1 | 0 | 0 |
|  |  |  | Cheese surface-Freshly prepared | 1 | 0 | 0 |
|  |  |  | Clean gallon of milk | 1 | 1 | 3 |
|  |  |  | Processing tank | 1 | 0 | 0 |
|  |  |  | Form for mold | 1 | 0 | 0 |
|  |  |  | Drain | 1 | 0 | 0 |
|  |  |  | Clean mold for cheese | 1 | 0 | 0 |
|  |  |  | Floor-Processing room | 1 | 0 | 0 |
|  |  |  | Wall-Processing room | 1 | 0 | 0 |
|  |  |  | Cheese strainer | 1 | 0 | 0 |
|  |  |  | Paddle to mix unpasteurized milk | 1 | 0 | 0 |
|  |  |  | Cloth in the soda boiler | 1 | 0 | 0 |
|  |  |  | Wall-Refrigerated room | 1 | 0 | 0 |
|  |  |  | Pallet-Refrigerated room | 1 | 0 | 0 |
|  |  |  | Mold from refrigerated room | 1 | 0 | 0 |
|  |  |  | Clean cloth | 1 | 0 | 0 |
|  |  |  | Handler's hand 1 | 1 | 0 | 0 |
|  |  |  | Handler's hand 2 | 1 | 0 | 0 |
|  |  |  | Pinking machine | 1 | 0 | 0 |
|  |  |  | Table for Minas cheese | 1 | 0 | 0 |
|  | Jul-14 | 30 | Brine | 1 | 0 | 0 |
|  |  |  | Unpasteurized milk | 1 | 1 | 2 |
|  |  |  | Pasteurized milk | 1 | 0 | 0 |
|  |  |  | Minas cheese surface 1 | 1 | 0 | 0 |
|  |  |  | Minas cheese 1 | 1 | 0 | 0 |
|  |  |  | Minas cheese surface 2 | 1 | 0 | 0 |
|  |  |  | Minas cheese 2 | 1 | 0 | 0 |
|  |  |  | Minas cheese surface 3 | 1 | 0 | 0 |
|  |  |  | Minas cheese 3 | 1 | 0 | 0 |
|  |  |  | Minas cheese 4 | 1 | 0 | 0 |
|  |  |  | Minas cheese surface 4 | 1 | 0 | 0 |
|  |  |  | Cheese surface -Freshly prepared | 1 | 0 | 0 |
|  |  |  | Recently prepared Minas cheese surface | 1 | 0 | 0 |
|  |  |  | Curd | 1 | 1 | 1 |
|  |  |  | Clean gallon of milk | 1 | 0 | 0 |
|  |  |  | Processing tank | 1 | 0 | 0 |
|  |  |  | Drain | 1 | 0 | 0 |
|  |  |  | Table for packing Minas cheese | 1 | 0 | 0 |
|  |  |  | Floor-Processing room | 1 | 1 | 1 |
|  |  |  | Wall-Processing room | 1 | 0 | 0 |
|  |  |  | Cheese strainer | 1 | 0 | 0 |
|  |  |  | Paddle to mix unpasteurized milk | 1 | 0 | 0 |
|  |  |  | Floor-Refrigerated room | 1 | 0 | 0 |
|  |  |  | Wall-Refrigerated room | 1 | 0 | 0 |
|  |  |  | Pallet -Refrigerated room | 1 | 0 | 0 |
|  |  |  | Mold of cheese from refrigerated room | 1 | 0 | 0 |
|  |  |  | Knife used to cut cheese | 1 | 0 | 0 |
|  |  |  | Handler's hand 1 | 1 | 0 | 0 |
|  |  |  | Handler's hand 2 | 1 | 0 | 0 |
|  |  |  | Table for the minas cheese | 1 | 0 | 0 |
| 4 | Dec-13 | 21 | Brine | 2 | 0 | 0 |
|  |  |  | Cheese surface | 2 | 0 | 0 |
|  |  |  | Cheese | 3 | 0 | 0 |
|  |  |  | Pasteurized milk | 1 | 0 | 0 |
|  |  |  | Glove | 1 | 0 | 0 |
|  |  |  | Drain | 4 | 0 | 0 |
|  |  |  | Surface (food-contact) | 8 | 0 | 0 |
|  | Jan-14 | 23 | Brine | 2 | 0 | 0 |
|  |  |  | Cheese surface | 4 | 0 | 0 |
|  |  |  | Cheese | 3 | 0 | 0 |
|  |  |  | Glove | 4 | 1 | 1 |
|  |  |  | Drain | 2 | 0 | 0 |
|  |  |  | Floor | 2 | 0 | 0 |
|  |  |  | Surface (food-contact) | 4 | 0 | 0 |
|  |  |  | Surface (non-food-contact) | 2 | 0 | 0 |
|  | Feb-14 | 53 | Brine | 2 | 0 | 0 |
|  |  |  | Cheese surface | 17 | 0 | 0 |
|  |  |  | Cheese | 4 | 0 | 0 |
|  |  |  | Pasteurized milk | 1 | 0 | 0 |
|  |  |  | Water | 1 | 0 | 0 |
|  |  |  | Glove | 8 | 1 | 4 |
|  |  |  | Drain | 3 | 0 | 0 |
|  |  |  | Floor | 2 | 0 | 0 |
|  |  |  | Surface (food-contact) | 13 | 0 | 0 |
|  |  |  | Surface (non-food-contact) | 2 | 0 | 0 |
|  | Mar-14 | 43 | Brine | 3 | 0 | 0 |
|  |  |  | Cheese surface | 6 | 0 | 0 |
|  |  |  | Cheese | 3 | 0 | 0 |
|  |  |  | Boot | 10 | 0 | 0 |
|  |  |  | Glove | 12 | 0 | 0 |
|  |  |  | Drain | 2 | 0 | 0 |
|  |  |  | Floor | 2 | 0 | 0 |
|  |  |  | Surface (food-contact) | 5 | 0 | 0 |
|  | Jul-14 | 28 | Brine | 2 | 0 | 0 |
|  |  |  | Cheese surface | 1 | 0 | 0 |
|  |  |  | Cheese | 3 | 0 | 0 |
|  |  |  | Pasteurized milk | 1 | 0 | 0 |
|  |  |  | Boots | 5 | 0 | 0 |
|  |  |  | Glove | 5 | 0 | 0 |
|  |  |  | Drain | 3 | 0 | 0 |
|  |  |  | Floor | 2 | 0 | 0 |
|  |  |  | Surface (food-contact) | 6 | 0 | 0 |
| 5 | Dec-13 | 30 | Brine | 1 | 0 | 0 |
|  |  |  | Curd | 1 | 0 | 0 |
|  |  |  | Cheese surface | 9 | 0 | 0 |
|  |  |  | Cheese | 2 | 0 | 0 |
|  |  |  | Pasteurized milk | 2 | 0 | 0 |
|  |  |  | Water | 1 | 0 | 0 |
|  |  |  | Glove | 2 | 0 | 0 |
|  |  |  | Floor | 2 | 0 | 0 |
|  |  |  | Drain | 2 | 0 | 0 |
|  |  |  | Surface (food-contact) | 8 | 0 | 0 |
|  | Jan-14 | 24 | Brine | 1 | 0 | 0 |
|  |  |  | Curd | 1 | 0 | 0 |
|  |  |  | Cheese surface | 6 | 0 | 0 |
|  |  |  | Cheese | 1 | 0 | 0 |
|  |  |  | Pasteurized milk | 1 | 0 | 0 |
|  |  |  | Water | 1 | 0 | 0 |
|  |  |  | Glove | 2 | 0 | 0 |
|  |  |  | Floor | 3 | 0 | 0 |
|  |  |  | Drain | 2 | 0 | 0 |
|  |  |  | Surface (food-contact) | 5 | 0 | 0 |
|  |  |  | Surface (non-food-contact) | 1 | 0 | 0 |
|  | Feb-14 | 24 | Brine | 1 | 0 | 0 |
|  |  |  | Curd | 1 | 0 | 0 |
|  |  |  | Cheese surface | 9 | 1 | 1 |
|  |  |  | Pasteurized milk | 1 | 0 | 0 |
|  |  |  | Glove | 1 | 0 | 0 |
|  |  |  | Drain | 2 | 0 | 0 |
|  |  |  | Floor | 2 | 0 | 0 |
|  |  |  | Surface (food-contact) | 7 | 0 | 0 |
|  | Mar-14 | 35 | Brine | 1 | 0 | 0 |
|  |  |  | Curd | 1 | 0 | 0 |
|  |  |  | Cheese surface | 13 | 0 | 0 |
|  |  |  | Pasteurized milk | 1 | 0 | 0 |
|  |  |  | Glove | 3 | 2 | 2 |
|  |  |  | Drain | 2 | 0 | 0 |
|  |  |  | Floor | 2 | 0 | 0 |
|  |  |  | Surface (food-contact) | 12 | 2 | 3 |
|  | Jul-14 | 25 | Brine | 1 | 0 | 0 |
|  |  |  | Curd | 1 | 0 | 0 |
|  |  |  | Cheese surface | 4 | 0 | 0 |
|  |  |  | Pasteurized milk | 1 | 0 | 0 |
|  |  |  | Boots | 2 | 0 | 0 |
|  |  |  | Glove | 2 | 0 | 0 |
|  |  |  | Drain | 2 | 0 | 0 |
|  |  |  | Floor | 2 | 0 | 0 |
|  |  |  | Surface (food-contact) | 8 | 0 | 0 |
|  |  |  | Surface (non-food-contact) | 2 | 0 | 0 |
| Total number | | |  | 421 | 31 | 66 |

## Supplementary Table 2

Characteristic of each *S. aureus* isolate identified in the five dairies between December 2013 and July 2014. Characteristics include dairy, sampling point, date, sequence type (ST), clonal complex (CC), antibiotic susceptibility profile, hemolytic activity, and toxin gene identification. The antibiotic susceptibility is defined as sensitivity (S), intermediate tolerance (I) and resistance (R) and was determined for all 11 antibiotics tested: Penicillin G (P), Erythromycin (E), Tetracycline (T), Clindamycin (Cl), Gentamicin (G), Ciprofloxacin (Ci), Vancomycin (V), Trimethoprim-Sulfamethoxazole (S), Chloramphenicol (Ca), Cefoxitin (Ce) and Bacitracin (B). Hemolytic activity was classified as α‐hemolysis (partial hemolysis), β‐hemolysis (complete hemolysis) or γ‐hemolysis (non‐hemolytic bacteria). The detection of four genes encoding enterotoxins (SE) found in SFP, the most two common SEA and SED, and also SEE and SEG.

| Dairy Date | | Sampling point | Isolate | ST | CC | Antibiotic susceptibility | | | | | | | | | | | Hemolysis | SE | | | |
| --- | --- | --- | --- | --- | --- | --- | --- | --- | --- | --- | --- | --- | --- | --- | --- | --- | --- | --- | --- | --- | --- |
|  |  |  |  |  |  | P | E | T | Cl | G | Ci | V | S | Ca | Ce | B |  | A | D | E | G |
| 2 | Feb-14 | Wall | Sa1 | 3531 | 1 | S | S | S | S | S | S | S | S | S | S | S | α | - | - | - | - |
|  |  | Wall | Sa2 | 3531 | 1 | S | S | S | S | S | S | S | S | S | S | S | α´ | - | - | - | - |
|  |  | Curd tub | Sa3 | 3531 | 1 | S | S | S | S | S | S | S | S | S | S | S | α´ | - | - | - | - |
|  |  | Cloth mold | Sa4 | 3531 | 1 | S | S | S | S | S | S | S | S | S | S | S | α´ | - | - | - | - |
|  |  | Cloth mold | Sa5 | 3531 | 1 | S | S | S | S | S | S | S | S | S | S | S | α | - | - | - | - |
|  |  | Cheese surface | Sa6 | 3531 | 1 | S | S | S | S | S | S | S | S | S | S | S | α´ | - | - | - | - |
|  |  | Cheese surface | Sa7 | 3531 | 1 | S | S | S | S | S | S | S | S | S | S | S | β | - | - | - | - |
|  |  | Sink | Sa8 | 3531 | 1 | S | S | S | S | S | S | S | S | S | S | S | γ | - | - | - | - |
|  |  | Sink | Sa9 | 3531 | 1 | S | S | S | S | S | S | S | S | S | S | S | α | - | - | - | - |
|  |  | Handler's hand | Sa11 | 3540 | 1 | S | S | S | S | S | S | S | S | S | S | S | β | - | - | - | - |
|  |  | Handler's hand | Sa12 | 3531 | 1 | S | S | S | S | S | S | S | S | S | S | S | α | - | - | - | - |
|  |  | Floor | Sa13 | 3531 | 1 | S | S | S | S | S | S | S | S | S | S | S | α´ | - | - | - | - |
|  |  | Floor | Sa14 | 3531 | 1 | S | S | S | S | S | S | S | S | S | S | S | α | - | - | - | - |
|  |  | Paddle | Sa15a clear | 3531 | 1 | S | S | S | S | S | S | I | S | S | S | S | β | - | - | - | - |
|  |  | Paddle | Sa15a opaque | 3531 | 1 | S | S | S | S | S | S | I | S | S | S | S | α | - | - | - | - |
|  |  | Paddle | Sa15b | 3531 | 1 | S | S | S | S | S | S | I | S | S | S | S | α | - | - | - | - |
|  |  | Paddle | Sa16a | 3531 | 1 | S | S | S | S | S | S | I | S | S | S | S | β | - | - | - | - |
|  |  | Paddle | Sa16b | 3531 | 1 | R | S | S | S | S | S | S | S | S | S | S | α´ | - | - | - | - |
|  |  | Pasteurized milk | Sa17 | 398 | 398 | R | R | S | S | S | S | S | S | S | S | S | β | - | - | - | - |
|  |  | Cheese surface | Sa18 | 3531 | 1 | S | S | S | S | S | S | S | S | S | S | S | α | - | - | - | - |
|  |  | Cheese surface | Sa19 | 3531 | 1 | S | S | S | S | S | S | I | S | S | S | S | β | - | - | - | - |
|  |  | Cheese surface | Sa20 | 3531 | 1 | S | S | S | S | S | S | S | S | S | S | S | α´ | - | - | - | - |
|  |  | Brine | Sa21 | 3531 | 1 | S | S | S | S | S | S | S | S | S | S | S | α´ | - | - | - | - |
|  |  | Brine | Sa22 | 3531 | 1 | S | S | S | S | S | S | S | S | S | S | S | α´ | - | - | - | - |
|  |  | Brine | Sa23 | 3531 | 1 | S | S | S | S | S | S | S | S | S | S | S | α | - | - | - | - |
|  |  | Cheese surface | Sa24 | 3531 | 1 | S | S | S | S | S | S | S | S | S | S | S | α´ | - | - | - | - |
|  |  | Cheese surface | Sa25 | 3531 | 1 | S | S | S | S | S | S | I | S | S | S | S | α´ | - | - | - | - |
|  |  | Cheese surface | Sa26 | 3531 | 1 | S | S | S | S | S | S | S | S | S | S | S | α | - | - | - | - |
|  |  | Cheese surface | Sa27 | 3531 | 1 | S | S | S | S | S | S | S | S | S | S | S | α | - | - | - | - |
|  |  | Processing tank | Sa28 | 3531 | 1 | S | S | S | S | S | S | S | S | S | S | S | α´ | - | - | - | - |
|  |  | Processing tank | Sa29a | 3531 | 1 | S | S | S | S | S | S | S | S | S | S | S | β | - | - | - | - |
|  |  | Processing tank | Sa29b | 3531 | 1 | S | S | S | S | S | S | I | S | S | S | S | β | - | - | - | - |
|  |  | Minas cheese | Sa30 | 3531 | 1 | S | S | S | S | S | R | I | S | S | S | S | α´ | - | - | - | - |
|  |  | Minas cheese | Sa31 | 3531 | 1 | S | S | S | S | S | S | S | S | S | S | S | α | - | - | - | - |
|  |  | Cheese surface | Sa33 | 3531 | 1 | S | S | S | S | S | S | S | S | S | S | S | α´ | - | - | - | - |
|  |  | Cheese surface | Sa34 | 3531 | 1 | S | S | S | S | S | S | S | S | S | S | S | α´ | - | - | - | - |
|  |  | Minas cheese | Sa35 | 3531 | 1 | S | S | S | S | S | S | S | S | S | S | S | α´ | - | - | - | - |
|  |  | Minas cheese | Sa38 | 3531 | 1 | S | S | S | S | S | S | S | S | S | S | S | α | - | - | - | - |
|  |  | Minas cheese | Sa39 | 3531 | 1 | S | S | S | S | S | S | I | S | S | S | S | α´ | - | - | - | - |
|  |  | Minas cheese | Sa40 | 3531 | 1 | S | S | S | S | S | S | I | S | S | S | S | β | - | - | - | - |
|  |  | Cheese surface | Sa42 | 3531 | 1 | S | S | S | S | S | S | S | S | S | S | S | α´ | - | - | - | - |
|  |  | Cheese surface | Sa43 | 3531 | 1 | S | S | S | S | S | S | I | S | S | S | S | α´ | - | - | - | - |
|  |  | Cheese surface | Sa44 | 3540 | 1 | R | S | I | S | S | S | I | S | S | S | S | β | - | - | - | - |
|  |  | Cheese surface | Sa45 | 3531 | 1 | S | S | S | S | S | S | S | S | S | S | S | β | - | - | - | - |
| 3 | Mar-14 | Unpasteurized milk | Sa46 | 1 | 1 | S | S | S | S | S | S | I | S | S | S | S | α´ | - | - | - | - |
|  |  | Unpasteurized milk | Sa47 | 1 | 1 | S | S | S | S | S | S | S | S | S | S | S | α´ | - | - | - | - |
|  |  | Unpasteurized milk | Sa48 | 126 | 126 | S | S | S | S | S | S | S | S | S | S | S | β | - | - | - | - |
|  |  | Unpasteurized milk | Sa49 | 1 | 1 | S | S | R | S | R | S | S | S | S | S | S | α´ | - | - | - | - |
|  |  | Clean gallon of milk | Sa50 | 3562 | 1 | S | S | S | S | S | S | I | S | S | S | S | β | - | - | - | - |
|  |  | Clean gallon of milk | Sa51 | 3562 | 1 | S | S | S | S | S | S | S | S | S | S | S | β | - | - | - | - |
|  |  | Clean gallon of milk | Sa52 | 3562 | 1 | S | S | S | S | S | S | S | S | S | S | S | β | - | - | - | - |
|  | Jul-14 | Unpasteurized milk | Sa58 | 97 | 97 | R | R | S | R | S | S | I | S | S | S | S | β | - | - | - | - |
|  |  | Unpasteurized milk | Sa59 | 97 | 97 | R | R | R | R | S | S | S | S | S | S | S | β | - | - | - | - |
|  |  | Floor | Sa67 | 5 | 5 | R | S | S | S | S | S | S | S | S | S | S | β | - | - | - | + |
|  |  | Curd | Sa74 | 1 | 1 | S | S | S | S | S | S | I | S | S | S | S | α´ | - | - | - | - |
| 4 | Jan-14 | Glove | BZ012 | 398 | 398 | R | R | S | S | S | S | S | S | S | S | S | β | - | - | - | - |
|  | Feb-14 | Glove | Sa106 | 188 | 1 | R | S | R | S | S | S | S | S | S | S | S | β | - | - | - | - |
|  |  | Glove | Sa107 | 3534 | 1 | R | R | R | R | S | S | I | S | S | S | S | β | - | - | - | - |
|  |  | Glove | Sa108 | 188 | 1 | R | S | R | S | S | S | S | S | S | S | S | β | - | - | - | - |
|  |  | Glove | Sa109 | 188 | 1 | R | R | R | R | S | S | I | S | S | S | S | β | - | - | - | - |
| 5 | Feb-14 | Cheese surface | Sa96 | 30 | 30 | R | S | S | S | S | S | S | S | S | S | S | β | + | - | - | + |
|  | Mar-14 | Glove | Sa101 | 97 | 97 | R | S | S | S | S | S | I | S | S | S | S | β | - | - | - | - |
|  |  | Glove | Sa102 | 30 | 30 | R | S | S | S | S | S | I | S | S | S | S | β | + | - | - | + |
|  |  | Bucket | Sa103 | 1 | 1 | S | S | S | S | S | S | I | S | S | S | S | β | - | - | - | - |
|  |  | Cold chamber shelf | Sa104 | 1 | 1 | S | S | S | S | S | S | S | S | S | S | S | β | - | - | - | - |
|  |  | Cold chamber shelf | Sa105 | 1 | 1 | S | S | S | S | S | S | S | S | S | S | S | β | - | - | - | - |
